# Successful Removal of an Entrapped Stent Delivery Catheter Using Two Arterial Sheaths in the Ipsilateral Groin

**DOI:** 10.7759/cureus.51138

**Published:** 2023-12-26

**Authors:** Eiji Miyauchi, Ryo Arikawa, Daisuke Tokutake, Naoya Oketani, Mitsuru Ohishi

**Affiliations:** 1 Department of Cardiology, Kagoshima City Hospital, Kagoshima, JPN; 2 Department of Cardiovascular Medicine and Hypertension, Graduate School of Medical and Dental Sciences, Kagoshima, JPN

**Keywords:** arterial sheath, double guide, stent delivery catheter, device entrapment, endovascular treatment (evt)

## Abstract

Entrapment of devices, such as a Rota bar, an extension catheter, or an intravascular ultrasound device, during percutaneous coronary intervention has been reported and bailout strategies have been discussed. However, there have been few reports on entrapment of devices during endovascular treatment (EVT). A 70-year-old man was referred to our clinic for the management of rest pain in his left lower limb. His left ankle-brachial index was unmeasurable and computed tomography angiography revealed total occlusion of the left common, external iliac, and superficial femoral arteries (SFA). He was diagnosed as having symptomatic limb-threatening ischemia and EVT was planned. The first EVT was performed on an occluding lesion in the left iliac artery. We used a transradial approach and deployed two bare nitinol stents in the left iliac artery without complications. One week after the first EVT, the second EVT was performed on an occluding lesion in the left SFA. A 6.0-French (Fr) guide sheath was inserted antegradely through the left common femoral artery. The occluded lesion was dilated with a 4.0 mm plain balloon, following which intravascular ultrasound revealed a localized severe stenotic lesion in the distal part of the SFA. A 6.0 mm drug-eluting stent was deployed to cover the stenotic lesion in the distal part of the SFA without pre-dilation; however, the stenotic lesion did not dilate sufficiently. When we attempted to extract the stent delivery catheter, we could not detach its tip from the localized severe stenotic lesion and were unable to remove it by force or external compression. Therefore, we decided to implement a double guide technique by inserting a 4.0-Fr sheath simultaneously into the left common femoral artery adjacent to the first puncture site together with another 0.014-inch guidewire via a 4.0-Fr sheath to get past the lesion in which the catheter tip was embedded. We then used a 3.0-mm plain balloon to dilate the severe stenotic lesion sufficiently to enable the removal of the stent delivery catheter. Another 6.0-mm drug-eluting stent was then deployed, after the first stent, to cover the occluded lesion in the middle part of the SFA. Hemostasis was safely achieved at both puncture sites by manual compression. A double guide technique, as in percutaneous coronary intervention, is useful for the bailout of an entrapped device during EVT. Careful consideration of the access site and size and length of the second guide sheath are necessary.

## Introduction

Entrapment of devices, such as a Rota bar, an extension catheter, or an intravascular ultrasound device, during percutaneous coronary intervention (PCI) has been reported [1] and bailout strategies have been discussed [2-4]. However, there have been few reports about managing the entrapment of devices during endovascular treatment (EVT).

Given that PCI and EVT are both endovascular interventions, techniques for managing the entrapment of devices during PCI are likely to be useful in EVT as well. However, in applying this knowledge, it is important to consider differences between EVT and PCI procedures, such as access sites and device sizes.

Here, we present our experience of successfully removing an entrapped stent delivery catheter by using two arterial sheaths in the ipsilateral groin.

## Case presentation

**Figure 1:**
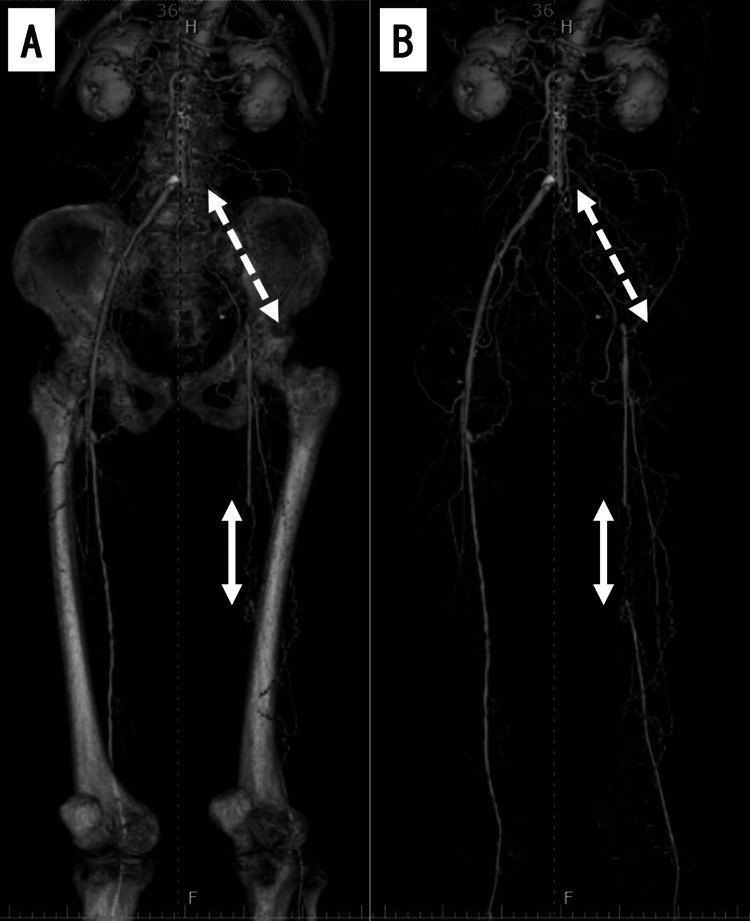
Three-dimensional computed tomography angiography of lower extremity arteries. (A) Image with bones. (B) Image without bones. These images showed complete occlusion of the left common iliac artery to the external iliac artery (white dotted arrow) and complete occlusion of the left superficial femoral artery (white arrow).

A 70-year-old man with a history of hypertension and cerebral infarction presented to our clinic for the management of rest pain in his left lower limb. He had psychroesthesia in his left foot, which appeared cyanotic. The common femoral, popliteal, dorsalis pedis, and posterior tibial arteries in his left lower limb were all impalpable. His left ankle-brachial index was unmeasurable. Computed tomography angiography revealed total occlusion of the left common, external iliac, and superficial femoral arteries (SFA) (Figure 1).

He was diagnosed as having chronic, symptomatic, limb-threatening ischemia, Rutherford class 4, and EVTs were planned.

**Figure 2:**
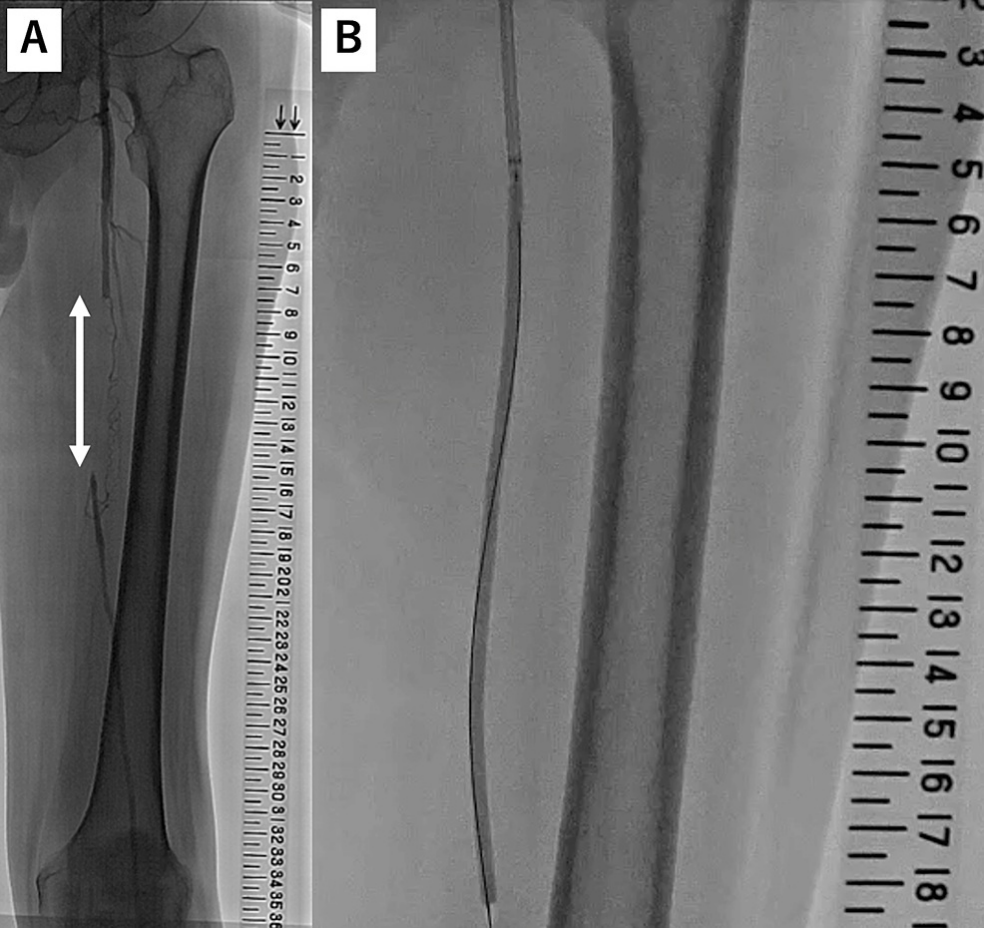
Angiography and fluoroscopic image of the left lower limb artery before and during endovascular treatment using plain balloon angioplasty. (A) Angiography of the left lower limb artery before endovascular treatment. An approximately 8 cm long completely occluded segment (white arrow) was detected in the middle portion of the superficial femoral artery. (B) Fluoroscopic image of the left lower limb artery during endovascular treatment using plain balloon angioplasty. The occluded lesion was dilated with a 4.0-mm plain balloon (Coyote 4.0/150 mm; Boston Scientific, Marlborough, MA, USA).

We used a transradial approach to perform the first EVT on an occluding lesion in the left iliac artery. Two bare nitinol stents were implanted in the left common and external iliac artery without complications. We performed the second EVT for an occluding lesion in the left SFA one week after the first EVT. Angiography revealed that the occlusion was approximately 80 mm long and in the middle segment of the SFA (Figure 2, Panel A).

**Figure 3:**
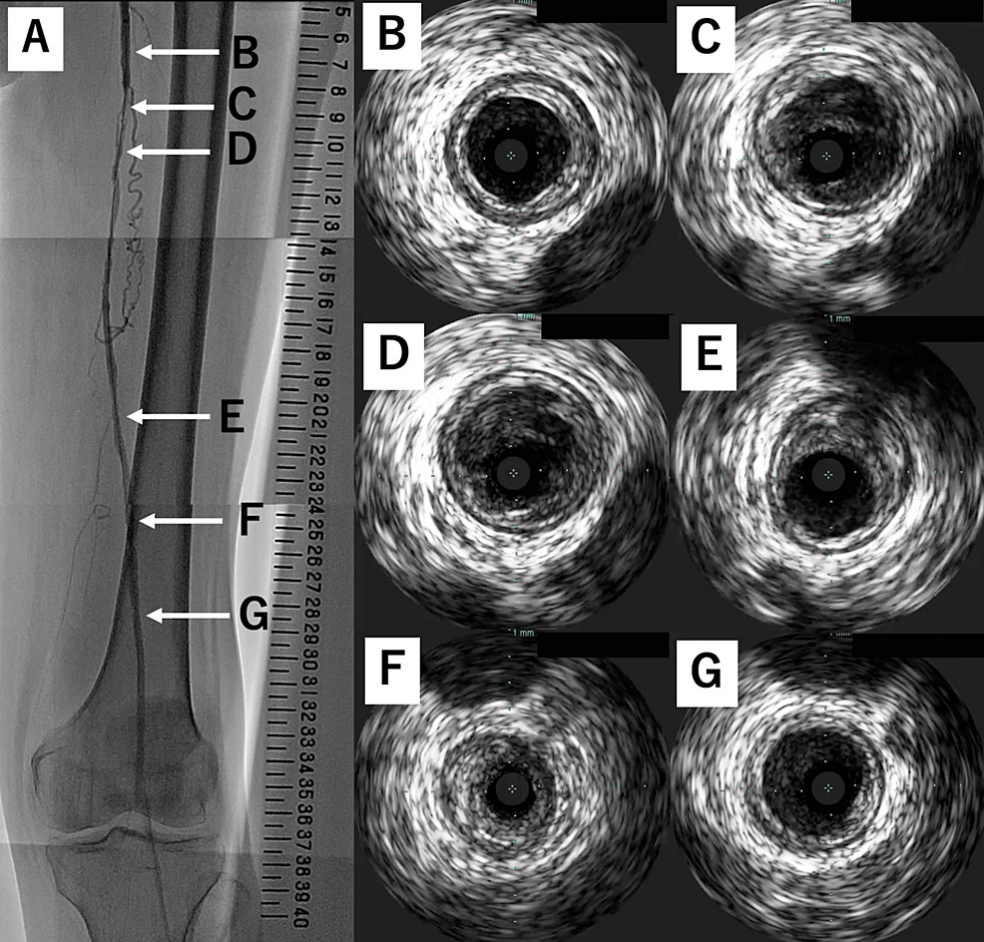
Catheter angiography and intravascular ultrasound images of the left lower limb obtained after pre-dilation. (A) Catheter angiography of the left lower limb. This image was created by merging several images into one and shows type D dissection in the middle part of the superficial femoral artery. (B-G) Intravascular ultrasound cross-sectional images from the proximal part of the superficial femoral artery to the middle part of the popliteal artery. (B) The vessel diameter is approximately 6.0 mm in the proximal reference site. (C-D) These segments are filled with thrombotic materials. (E) Mild stenosis with fibrous plaque. (F) Severe stenosis with extensive fibrous plaque. (G) The vessel diameter is approximately 6.0 mm at the distal reference site.

We inserted a 6.0-French (Fr) guide sheath (Parent Plus 21 cm; Medikit, Tokyo, Japan) antegradely via the left common femoral artery and successfully passed a 0.014-inch guidewire (Jupitar FC 235 cm; Boston Scientific, Marlborough, MA, USA) supported by a penetration catheter (Corsair PV 135 cm; Asahi Intecc, Tokyo, Japan) through the occluded lesion. Because intravascular ultrasound could not pass through the occluded lesion without pre-dilation, we speculated non-compliant balloons might not pass the occluded lesion, and the lesion was dilated with a 4.0-mm semi-compliant balloon (Coyote 4.0/150 mm; Boston Scientific, Marlborough, MA, USA) (Figure 2, Panel B). Angiography then showed type D dissection in the occluded lesion (Figure 3, Panel A). Intravascular ultrasound revealed that the lumen of the occluded vessel contained thrombotic material and that the stenosis in the distal segment of SFA was particularly severe at 25 on the scale (Figure 3).

**Figure 4:**
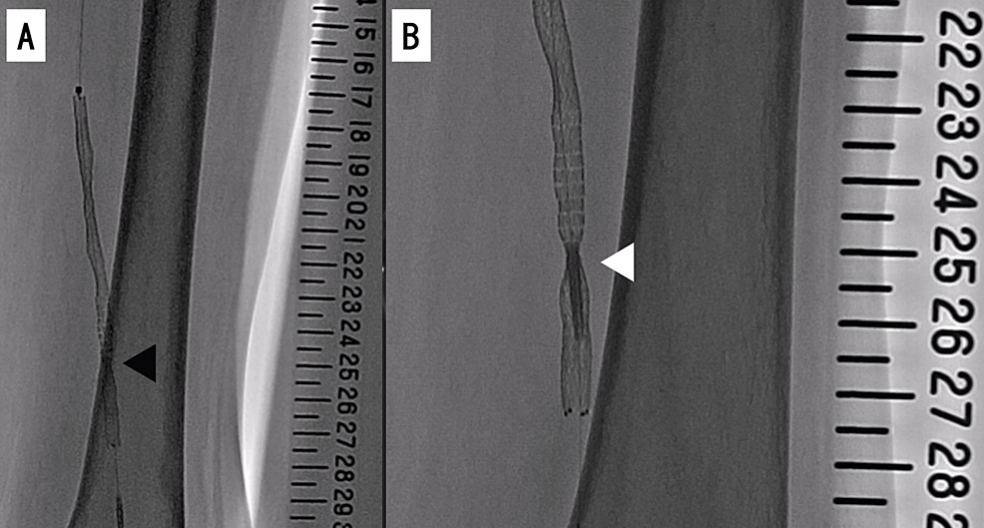
Fluoroscopy of the distal part of the superficial femoral artery after stent deployment. (A) Immediately after stent deployment, there is still indentation of the stent (black arrowhead) at 25 on the scale. (B) When we tried to extract the stent delivery catheter, we could not detach the tip of the catheter from the severe localized stenotic lesion at 25 on the scale (white arrowhead).

To minimize the potential for distal embolization, we attempted to place drug-eluting stents to cover the occluding and stenotic lesions without performing additional pre-dilation. However, the lesion that was 25 on the scale was not sufficiently dilated during deployment of the first stent (Eluvia 6.0/120 mm; Boston Scientific, Marlborough, MA, USA) (Figure 4, Panel A).

**Figure 5:**
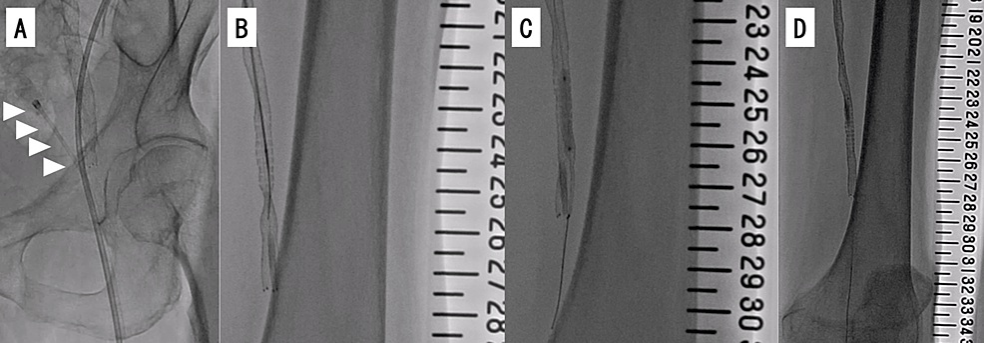
Bailout procedure for entrapped stent delivery catheter shaft using a double guide catheter technique. (A) A 4-Fr sheath (white arrowheads) was antegradely inserted into the left common femoral artery. (B) The insufficiently dilated site was bypassed with a 0.014-inch guidewire. (C) The insufficiently dilated site was dilated with a 3.0-mm plain balloon. (D) The site is no longer indented. We then succeeded in removing the stent delivery catheter shaft.

When we attempted to extract the stent delivery catheter, we could not detach its tip from the localized severe stenotic lesion and were unable to remove it by force or external compression (Figure 4, Panel B). Next, we tried the following double guide technique. We simultaneously inserted a 4.0-Fr sheath into the left common femoral artery adjacent and superior to the first puncture site (Figure 5, Panel A) and another 0.014-inch guidewire (Command 250 mm; Abbot, Chicago, IL, USA) via a 4.0 Fr sheath to bypass the lesion in which the catheter tip was embedded (Figure 5, Panel B).

**Figure 6:**
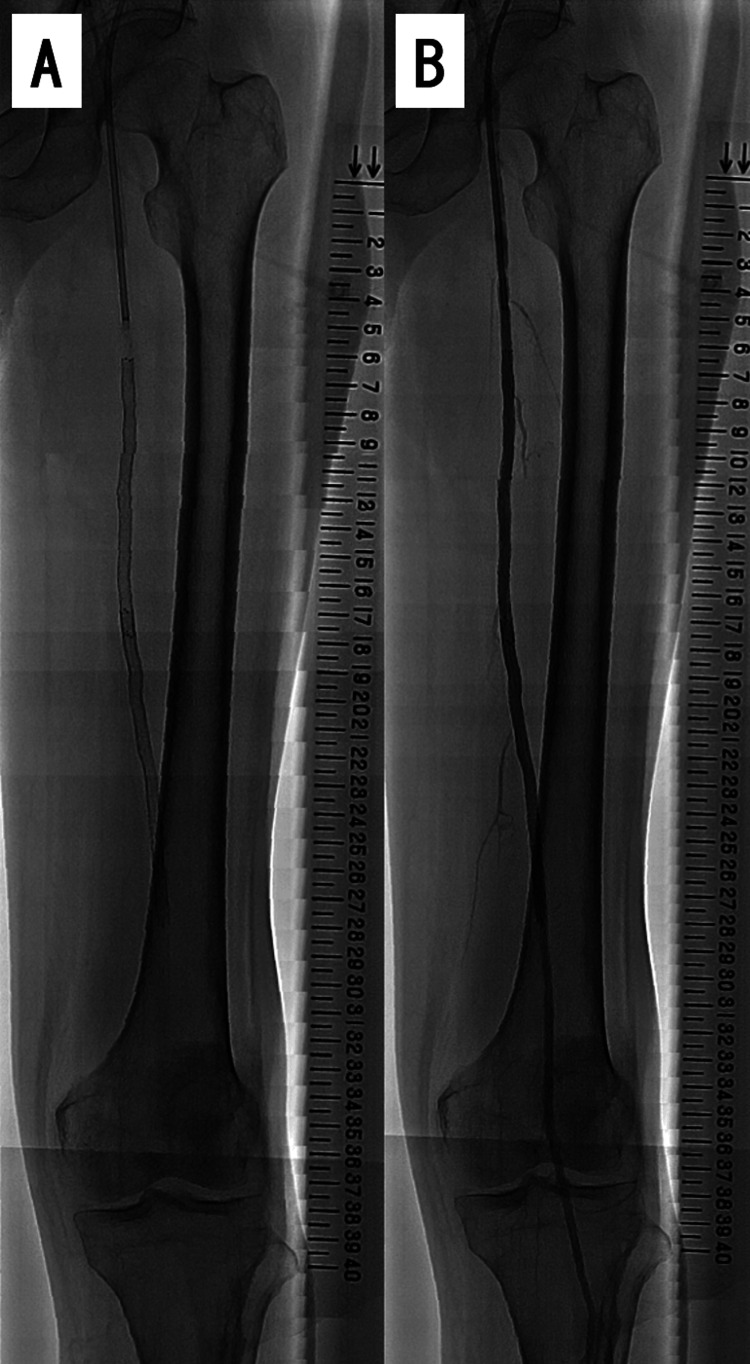
Fluoroscopy and angiography after endovascular treatment. (A) Fluoroscopy shows stents have been deployed from 6 through 27 on the scale. (B) Final angiography image showing improvement in the lesion with stent deployment.

We then used a 3.0-mm semi-compliant balloon (Coyote 3.0 /20 mm, Boston Scientific, Marlborough, MA, USA) to dilate the severe stenotic lesion sufficiently (Figure 5, Panel C) to enable the removal of the stent delivery catheter. Next, we deployed another 6.0-mm drug-eluting stent (Eluvia 6.0/120 mm; Boston Scientific, Marlborough, MA, USA) to cover the occluding lesion in the middle part of the SFA. We then dilated both stents further with a smaller balloon (Coyote 4.0/150 mm; Boston Scientific, Marlborough, MA, USA) to prevent distal embolization. Angiography after EVT showed complete filling of the SFA (Figure 6).

Hemostasis was safely achieved at both puncture sites with manual compression. After the EVT, our patient’s left ankle-brachial index improved from unmeasurable to 1.01 and the symptoms in his left leg resolved.

## Discussion

No bailout strategy for device entrapment during EVT has yet been established. In the present case, the stent delivery catheter got trapped in stent struts that were not fully dilated. Having found no reports of such cases, we devised the following bailout strategy.

Forcible withdrawal of trapped devices, such as stent delivery catheter shafts or stent struts, should be avoided because this may cause device fracture. Device fracture necessitates retrieval procedures, which may lead to both failure of revascularization and serious complications such as vascular injury or thrombosis [5].

In the field of PCI, a double guide catheter technique has been reported as a bailout strategy for entrapped devices [3,4,6-8]. This strategy enables the utilization of new devices, such as guidewires, catheters, and balloons, via a second guide sheath and can enable retrieval of entrapped devices. Several factors must be considered when employing a double guide catheter technique during EVT, including the direction of approach (antegrade or retrograde), the side of approach (contralateral or ipsilateral), and the size and length of the guide sheath, which varies depending on the specific device being used. In our case, we adopted an antegrade ipsilateral approach via the common femoral artery because of the following three benefits. First, this approach allowed us to perform the puncture without changing the patient’s body position. Second, this access site was close to the catheter embedded in the lesion. Third, the common femoral artery was large enough to insert a larger sheath if required; however, careful attention was necessary during hemostasis.

A double guide catheter technique has been reported as a useful bailout strategy for vessel rupture during PCI [6,7]; thus, a similar technique could also be useful during EVT. An antegrade approach would be useful if vessel rupture occurred because this approach would enable getting better angiography images and deploying a covered stent, which requires a 7-8-Fr guide sheath.

Next, avoidance of device entrapment is crucial. Compared with balloon-expandable stents, self-expandable stents occasionally fail to create insufficient expansion, especially in the acute phase. Thus, it is important to use an imaging modality to evaluate the pre-dilation before deploying a stent, especially in the lesion with severe calcification or a high burden of fibrous plaque. In our case, we avoided additional pre-dilation of the proximal lesion because it was thrombotic. However, given that the distal lesion contained fibrous plaque, which imposes a high burden on intravascular ultrasound, additional pre-dilation of the distal lesion would have been advisable. Furthermore, for a larger acute gain, balloon dilation using a non-compliant balloon should have been performed. When fibrous lesions and thrombotic plaques coexist, as was true in our case, the balloon size for pre-dilation should be carefully selected. Moreover, the use of distal protection devices should also be considered.

## Conclusions

Drug-eluting stent was deployed to cover the stenotic lesion in the distal part of the SFA without pre-dilation. When we attempted to extract the stent delivery catheter, we could not detach its tip from the localized severe stenotic lesion and were unable to remove it by force or external compression. A double guide catheter technique, as has been described for PCI, proved useful for the bailout of an entrapped device during EVT. Careful consideration of the access site and the size and length of a second guide sheath were necessary.
